# Evaluation of the Accuracy of Responses Provided by AI-Based Conversational Systems to Patient Questions Regarding Endodontic Pain and Antibiotic Use

**DOI:** 10.7759/cureus.107212

**Published:** 2026-04-17

**Authors:** Hazal Faiz Arslanparcasi, Mehmet Ali Arslanparcasi

**Affiliations:** 1 Endodontics, Harran University, Sanliurfa, TUR; 2 Dental Prosthesis Technology, Faculty of Vocational School of Health Services Harran University, Sanliurfa, TUR

**Keywords:** antibiotic use, artificial intelligence, conversational systems, endodontic pain, patient education

## Abstract

Aim

This study aimed to comparatively evaluate the accuracy of responses provided by different AI-based conversational systems to patient questions regarding endodontic pain and antibiotic use.

Methods

In this study, a total of 20 clinical scenarios related to endodontic pain and antibiotic use were prepared. Ten of the scenarios represented clinical conditions in which antibiotic use was indicated, whereas the other 10 represented conditions in which it was not indicated. All prepared scenarios were directed to four different AI-based systems: ChatGPT (OpenAI, San Francisco, USA), DeepSeek (DeepSeek AI, Hangzhou, China), Gemini (Google, Mountain View, USA), and Copilot (Microsoft, Redmond, USA), and responses were recorded by initiating a new session for each scenario in the relevant system. The responses were evaluated by an endodontic specialist using a 3-point scale in terms of antibiotic use indications (1 = incorrect, 2 = partially correct, 3 = correct). Wilcoxon signed-rank test and Kruskal-Wallis test were used for data analysis, and the significance level was set at p < 0.05.

Results

All AI systems showed similar performance in scenarios where antibiotic use was indicated and not indicated. The difference between indicated and non-indicated scenarios was not statistically significant for ChatGPT, DeepSeek, Gemini, and Copilot (p = 0.317, p = 0.564, p = 0.317, and p = 0.102, respectively). No significant difference was also found among the AI systems in terms of overall performance (H = 3.292; p = 0.349). As each of the 20 clinical scenarios was submitted to four different AI-based conversational systems, a total of 80 responses were evaluated. Of these, 56 were classified as correct and 24 as partially correct, whereas no responses were observed in the incorrect category.

Conclusion

The evaluated AI-based conversational systems generally provided correct or partially correct responses to patient questions related to endodontic pain and antibiotic use. No statistically significant difference was found among the systems, and all systems demonstrated similar performance. These findings suggest that AI-based systems may have supportive potential in patient information provision. Nevertheless, due to the presence of incomplete or ambiguous responses, it is clear that these systems should not replace expert evaluation.

## Introduction

The use of antibiotics in the treatment of endodontic infections is limited, and the primary approach consists of local dental interventions aimed at eliminating the source of infection; systemic antibiotics are indicated only in specific clinical situations [1]. Particularly in conditions such as acute pulpitis and symptomatic apical periodontitis, pain management is generally limited to aseptic removal of the pulp and the use of analgesic adjuncts, and antibiotic molecules do not possess direct pain control mechanisms [2]. The European Society of Endodontology guidelines provide evidence-based criteria for antibiotic use in the treatment of endodontic infections [3].

Nevertheless, antibiotics may in some cases be prescribed by dentists in suboptimal or non-indicated clinical situations [4]. When faced with symptoms such as dental pain, patients tend to seek health-related information online, particularly through AI-based conversational systems. Public interaction with dental misinformation on social media has been documented and may amplify the impact of inaccurate automated responses [5]. As the popularity of these systems increases, the reliability and accuracy of the information they provide to users become critically important. Incorrect or incomplete information regarding antibiotic use may negatively affect patients’ health outcomes by leading to unnecessary use, delayed professional dental treatment, and misinterpretation of urgent clinical conditions [6]. In this context, the number of comprehensive studies examining the accuracy of information provided by AI systems on sensitive topics such as endodontic pain management and antibiotic indications remains quite limited [7,8].

AI-based conversational systems such as ChatGPT (OpenAI, San Francisco, USA), Gemini (Google, San Francisco, USA), Copilot (Microsoft, Redmond, USA), and DeepSeek (DeepSeek AI, Hangzhou, China) have increasingly become common tools for accessing health information by generating responses in natural language [9,10]. Although existing studies evaluating the accuracy and consistency of the responses provided by these systems to questions related to endodontics have generally focused on general endodontic knowledge [11,12], they do not sufficiently address specific and critical issues such as indications for antibiotic use. This situation necessitates closer examination of the potential impact of the information patients obtain from these systems and its implications for public health, particularly in light of global health problems such as antibiotic resistance [13].

This study aimed to comparatively evaluate the accuracy of responses provided by different AI-based conversational systems to patient questions regarding endodontic pain and antibiotic use. The primary outcome of the study was the accuracy of AI-generated responses in terms of consistency with guideline-based antibiotic indications, whereas the secondary outcome was the comparison of response performance among the evaluated AI-based conversational systems.

## Materials and methods

Study design

This study was designed as a comparative observational study that evaluated the accuracy of responses provided by different AI-based conversational systems to patient questions regarding endodontic pain and antibiotic use.

As this study did not involve human participants, patient data, or biological samples, ethical approval was not required.

Preparation of the question set

Questions related to endodontic pain and antibiotic use were prepared by an instructor (MAA). The questions were developed with reference to the European Society of Endodontology (ESE) position statement and the evidence-based clinical practice guidelines published by the American Dental Association (ADA).

A total of 20 clinical scenarios were prepared (Table 1). Ten scenarios represented clinical conditions in which antibiotic use was indicated. Ten scenarios represented clinical conditions in which antibiotic use was not indicated.

**Table 1:** Clinical scenarios related to endodontic pain and antibiotic use, with predefined antibiotic indication status.

#	Patient Complaint	Antibiotic Requirement
1	“I have mild throbbing in my tooth, but I do not have facial swelling or fever. Do I need to take antibiotics?”	No
2	“My tooth has started throbbing, my cheek is swelling, and I have a fever. I have a congenital heart defect. Would my doctor prescribe antibiotics?”	Yes
3	“Pus is draining from my lower tooth, and my jaw feels stiff, and I cannot open it. Do I need to use antibiotics?”	Yes
4	“I have throbbing pain in my tooth, my fever is above 38°C, and I have chills. Would antibiotics be prescribed?”	Yes
5	“I have occasional tooth pain that goes away with painkillers. Are antibiotics necessary?”	No
6	“My upper molar hurts badly, swelling has developed, and the glands in my neck are also swollen. Should I take antibiotics?”	Yes
7	“I have mild throbbing in my tooth, and my jaw feels stiff, but there is no swelling or fever. Are antibiotics necessary?”	No
8	“Pus is draining from my upper molar, and my jaw feels stiff, and I cannot open it. Are antibiotics necessary?”	Yes
9	“I have a mild localized abscess in my tooth, but I do not have swelling or fever. Should I take antibiotics?”	No
10	“I have firm swelling in my tooth area, severe pain, and redness on my face. I have received intravenous bisphosphonate therapy. Would antibiotics help?”	Yes
11	“Pus is draining from my lower molar, and I have a mild fever and swelling. I am receiving chemotherapy. Are antibiotics necessary?”	Yes
12	“I have tooth sensitivity, but there is no swelling, fever, or swollen neck lymph nodes. Should I take antibiotics?”	No
13	“My upper molar is sensitive to hot and cold, but my gums look normal. Would my doctor prescribe antibiotics?”	No
14	“My abscess is growing rapidly, and there is hardness and redness in my cheek. I am receiving treatment for leukemia. Are antibiotics necessary?”	Yes
15	“My lower tooth hurts, but I do not have facial swelling or fever. Are antibiotics necessary?”	No
16	“My tooth that had root canal treatment hurts slightly, but my gums look normal. Are antibiotics necessary?”	No
17	“I have tooth pain that increases with heat, but there is no swelling or fever in my jaw. Are antibiotics necessary?”	No
18	“I have a rapidly spreading abscess, increasing facial swelling, and a risk of endocarditis. I also have a fever. Should I use antibiotics?”	Yes
19	“My upper molar is throbbing, the pain has increased, and I have swelling. I have a prosthetic heart valve. Should I take antibiotics?”	Yes
20	“My upper molar is sensitive, my gums look normal, and I have no systemic symptoms. Would my doctor prescribe antibiotics?”	No

The clinical scenarios were constructed to reflect real patient language, and the correct response for each scenario was predetermined according to the guidelines. The clinical scenarios were intentionally constructed in patient-oriented language to reflect real-world patient questions, and the correct response for each scenario was predetermined according to the guidelines. 

Data collection process

All prepared clinical scenarios were directed to four different AI-based systems: ChatGPT 5.4, Copilot (web version), Gemini 3, and DeepSeek (web version). All queries were performed on March 29, 2026. As AI-based systems are continuously updated, the responses reflect the versions accessible on the respective platforms on that date. For each clinical scenario, a new session was initiated in the relevant AI system in order to minimize the effect of previous responses. The obtained responses were recorded without any modification and archived for analysis.

Evaluation process

The responses were evaluated by an endodontic specialist (HFA) in terms of indications for antibiotic use. A 3-point scale was used in the evaluation process, where 1 = Incorrect (recommending unnecessary antibiotic use or failing to recommend it when indicated), 2 = Partially correct (incomplete or ambiguous response), 3 = Correct (guideline-consistent response).

Responses were classified according to their compatibility with the relevant clinical guidelines. Responses that were fully consistent with the guideline and contained sufficient information were classified as correct, those containing incomplete or ambiguous information were classified as partially correct, and those inconsistent with the guideline were classified as incorrect. The evaluation was based on predefined guideline-derived criteria, developed with reference to the European Society of Endodontology (ESE) position statement and the evidence-based clinical practice guideline published by the American Dental Association (ADA) [14,15], rather than individual clinical preference. Responses were scored using a three-point scale, and the evaluator was blinded to system identity during the assessment process.

Statistical analysis

As the data did not show a normal distribution, non-parametric tests were used. The Wilcoxon signed-rank test was applied to compare the performance of the AI systems in scenarios where antibiotic use was indicated and not indicated. Within the scope of this analysis, mean scores and standard deviations were calculated for indicated and non-indicated scenarios for each AI system.

The Kruskal-Wallis test was used to compare the different AI systems, and the level of significance was set at p < 0.05 for all analyses. All statistical analyses were performed using SPSS version 27 (IBM Corp., Armonk, USA).

## Results

Within-system performance of AI systems

The responses provided by four different AI systems (ChatGPT, DeepSeek, Gemini, and Copilot) to questions related to endodontic pain and antibiotic use were evaluated using the Wilcoxon signed-rank test in terms of scenarios in which antibiotic use was indicated and not indicated. Mean scores and standard deviations for indicated and non-indicated scenarios for each AI system are presented in Table 2.

**Table 2:** Mean scores and standard deviations of AI systems in scenarios where antibiotic use was indicated and not indicated (Wilcoxon test).

AI System	Antibiotic Indicated (Mean ± SD)	Antibiotic Not Indicated (Mean ± SD)	p-value
ChatGPT	2.9 ± 0.32	2.8 ± 0.42	0.317
DeepSeek	2.7 ± 0.53	2.6 ± 0.54	0.564
Gemini	2.8 ± 0.42	2.6 ± 0.52	0.317
Copilot	2.8 ± 0.42	2.4 ± 0.52	0.102

The mean response scores of ChatGPT were 2.9 ± 0.32 in scenarios where antibiotic use was indicated and 2.8 ± 0.42 in scenarios where antibiotic use was not indicated. The Wilcoxon test results showed p = 0.317 (p > 0.05), indicating no statistically significant difference. Equal scoring was observed in seven of the 10 questions; indicated scenarios received higher scores in two questions, whereas non-indicated scenarios received higher scores in one question. This finding suggests that ChatGPT showed similar performance in scenarios where antibiotic use was indicated and not indicated.

For DeepSeek, the mean scores for scenarios where antibiotic use was indicated and not indicated were 2.7 ± 0.53 and 2.6 ± 0.54, respectively, and the Wilcoxon test yielded p = 0.564 (p > 0.05). Equal scoring was observed in seven of the 10 questions; indicated scenarios received higher scores in two questions, whereas non-indicated scenarios received higher scores in one question. No statistically significant difference was found between DeepSeek’s performance in scenarios where antibiotic use was indicated and not indicated.

Gemini’s mean response scores were 2.8 ± 0.42 for scenarios where antibiotic use was indicated and 2.6 ± 0.52 for scenarios where antibiotic use was not indicated. The Wilcoxon test yielded p = 0.317 (p > 0.05), indicating no statistically significant difference. Equal scoring was observed in six of the 10 questions; scenarios where antibiotic use was indicated received higher scores in three questions, whereas scenarios where antibiotic use was not indicated received higher scores in one question. Gemini was observed to demonstrate similar performance in scenarios where antibiotic use was indicated and not indicated.

For Copilot, the mean response scores for scenarios where antibiotic use was indicated and not indicated were 2.8 ± 0.42 and 2.4 ± 0.52, respectively, and the Wilcoxon test yielded p = 0.102 (p > 0.05). Equal scoring was observed in four of the 10 questions; indicated scenarios received higher scores in five questions, whereas non-indicated scenarios received higher scores in one question. Although greater variability was observed in Copilot, this difference was not statistically significant.

**Figure 1:**
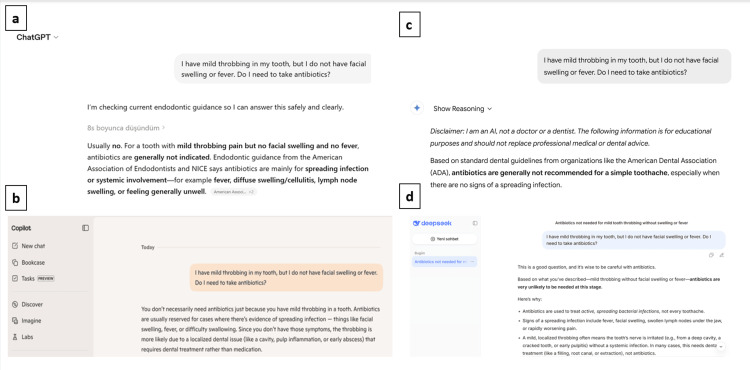
Responses provided by (a) ChatGPT, (b) Copilot, (c) Gemini, and (d) DeepSeek to the same clinical question.

The responses provided by ChatGPT, Copilot, Gemini, and DeepSeek to the same clinical question are presented in Figure 1. Overall, the AI systems showed similar performance in scenarios where antibiotic use was indicated and not indicated. Although relatively greater variability was observed in Copilot, this difference was not statistically significant.

Performance comparison among AI systems

The Kruskal-Wallis test was applied to evaluate differences in performance among the responses provided by four AI systems (ChatGPT, DeepSeek, Gemini, and Copilot) across all questions. As each of the 20 clinical scenarios was submitted to four different AI-based systems, a total of 80 responses were included in the analysis. As a result of the test, the total sample size was found to be n = 80, the test statistic was H = 3.292, the degree of freedom was df = 3, and the asymptotic significance value was p = 0.349 (Table 3). 

**Table 3:** Performance comparison among AI systems (Kruskal-Wallis test).

Test	Total N	Test Statistic (H)	Degrees of Freedom (df)	Asymptotic Sig. (p)
Kruskal-Wallis	80	3.292	3	0.349

Since the p-value was greater than 0.05, the null hypothesis could not be rejected; no statistically significant difference was found among the AI systems. The score distributions of the four AI systems across the questions were found to be similar. Therefore, pairwise post hoc comparisons were not performed.

**Figure 2:**
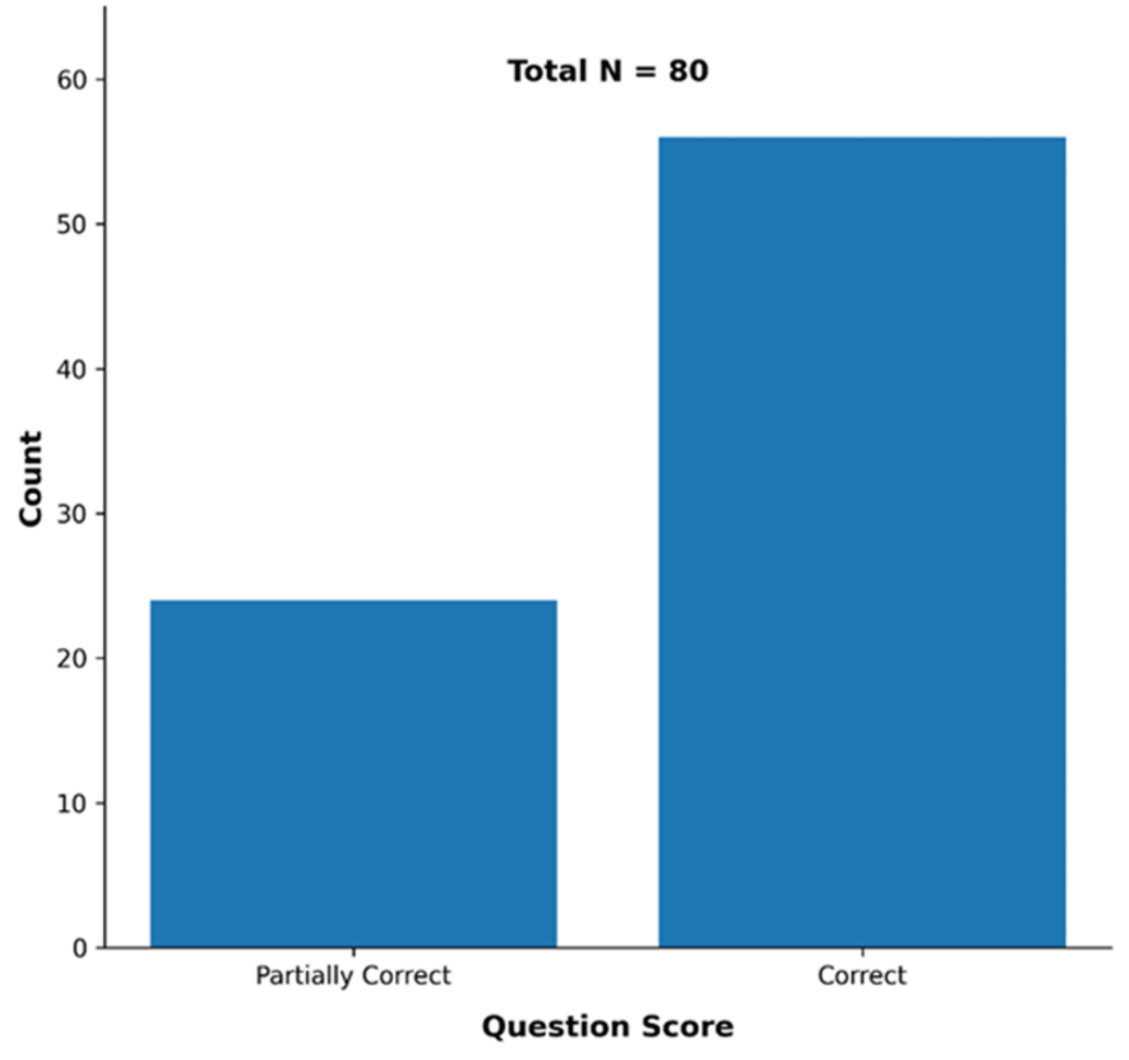
Categorical distribution of question scores.

Upon examination of the categorical distribution of question scores, 56 of the total 80 evaluations were classified as “correct,” whereas 24 were classified as “partially correct.” No responses were observed in the “incorrect” category. This distribution indicates that the majority of the responses provided by the AI systems were consistent with the guidelines, while the remaining responses contained incomplete or ambiguous information (Figure 2).

## Discussion

In this study, the accuracy and completeness of responses provided by AI-based conversational systems to patient questions related to endodontic pain and antibiotic use were evaluated [16]. The findings obtained showed that the systems examined generally had a high level of accuracy. Of the total 80 evaluations, 56 were classified as correct and 24 as partially correct, and no responses were observed in the incorrect category. In addition, no statistically significant difference in performance was detected among the AI systems, and similar performance was obtained in scenarios where antibiotic use was indicated and not indicated. These findings suggest that AI-based conversational systems have potential in patient information provision, although there are still aspects that need improvement in terms of providing complete and consistent information. In particular, our study showed that AI responses demonstrated similar performance in critical issues such as conditions requiring endodontic treatment and indications for antibiotic use; however, there are still areas for improvement in terms of accuracy and completeness [12].

These results obtained in our study are generally consistent with previous studies investigating the accuracy of information provided by AI-based language models in specific and sensitive fields such as dentistry [16]. For example, it has been reported that large language models generally perform well on knowledge-based questions, although differences may exist in clinical sciences [17]. Some studies have indicated that the quality of responses provided by AI chatbots to patient questions may be superior to that of human experts, particularly for short and standardized questions, and that these systems may serve as an important supportive tool [18]. However, when specific clinical scenarios and complex cases are involved, inconsistencies and errors may be observed in the responses of these systems [19]. On the other hand, while some studies have shown that AI-based models can achieve accuracy rates of up to 80% in dental trauma [20] and general dentistry topics, another study emphasized that this technology is not yet fully reliable in clinical scenarios [21].

These findings demonstrate that AI-based systems require further improvement and validation for integration into areas such as diagnosis and treatment planning in dentistry [22]. Indeed, systematic reviews on the effectiveness of AI models in various endodontic applications, such as endodontic treatment evaluation, periapical lesion detection, and assessment of root canal filling, have revealed their promising potential in evaluating diagnostic accuracy and treatment outcomes [23]. However, it has been reported that large language models have limited clinical use in endodontic diagnosis and that improvements are needed in diagnostic accuracy in dentistry [24]. Although the potential of AI in endodontics is high, this indicates that more comprehensive validation and development processes are required before its integration into clinical practice [25]. It should also be taken into consideration that AI models may provide static responses during their learning processes and that their potential for updating over time may affect their performance [26]. This situation highlights the importance of current and dynamic health information and indicates that the ability of AI-based systems to adapt through continuous updating with current scientific data should be improved [27].

Therefore, in the applications of AI in dentistry, especially in subjects requiring patient information provision, such as endodontic pain and antibiotic use, it is of great importance that these systems be continuously updated with current scientific literature and that their clinical accuracy be verified by independent mechanisms [28,29]. In addition, further studies are needed to improve the diagnostic accuracy and treatment planning capabilities of AI-based systems in endodontic treatment [30]. In this regard, overcoming disadvantages such as limited data accessibility, lack of rigor in development, and ethical concerns is critical for broader and more successful use in diagnostic and treatment processes in dental clinics.

In this context, evaluating the accuracy of responses provided by AI-based conversational systems to patient questions related to endodontic pain and antibiotic use is important for determining the potential role and current limitations of these systems in clinical information provision. These analyses provide valuable insights into how AI applications in endodontics may improve diagnostic accuracy and treatment planning.

The present study has several limitations. First, it was conducted with a limited number of clinical scenarios and a limited number of AI-based systems, which may restrict the generalizability of the findings. Second, the clinical scenarios were predefined and may not fully reflect the complexity and variability of real patient questions encountered in daily practice, which may have limited the external validity of the study. Third, the responses were evaluated by a single endodontic specialist, which may have introduced evaluator-related bias. In addition, the assessment was not blinded, which may have increased the risk of subjective interpretation. Furthermore, AI-based systems are dynamic and continuously updated; therefore, the responses obtained on March 29, 2026, may differ from those generated at other time points, which limits reproducibility over time. Future studies with larger scenario sets, multiple evaluators, blinded assessment, and repeated evaluations at different time points may provide more robust evidence. In addition, the findings may have been influenced by model versioning and prompt sensitivity, as even small changes in platform updates or prompt phrasing may affect AI-generated responses.

Overall, the findings of this study indicate that AI-based conversational systems may play a supportive role in patient information provision regarding endodontic pain and antibiotic use; however, it should not be forgotten that these systems cannot replace expert evaluation in the clinical decision-making process.

## Conclusions

Within the limitations of this study, the evaluated AI-based conversational systems generally produced responses that were classified as correct or partially correct in relation to guideline-based antibiotic indications. However, these findings should be interpreted cautiously, given the limited scenario set, single-evaluator design, and the possibility that the absence of incorrect ratings may reflect the scoring approach rather than perfect model performance. Therefore, these systems may serve as supportive informational tools but should not replace expert clinical evaluation.
